# Supplementary material for: Intragenomic sequence variations in the second internal transcribed spacer (ITS2) ribosomal DNA of the malaria vector Anopheles stephensi
Source: PLoS One. 2021 Jun 14;16(6):e0253173. doi: 10.1371/journal.pone.0253173 (PMC8202910; doi:10.1371/journal.pone.0253173)
Supplement: S1 File — (PDF) [file pone.0253173.s001.pdf]

Supplementary information

S1: Annotation features of GenBank entries

```
>Feature gb|MW676288
<1    52         5.8S ribosomal RNA
53    522        internal transcribed spacer 2
523   >1679      28S ribosomal RNA

>Feature gb|MW676289
<1    52         5.8S ribosomal RNA
53    520        internal transcribed spacer 2
521   >1677      28S ribosomal RNA

>Feature gb|MW676290
<1    52         5.8S ribosomal RNA
53    520        internal transcribed spacer 2
521   >1677      28S ribosomal RNA

>Feature gb|MW676291
<1    52         5.8S ribosomal RNA
53    520        internal transcribed spacer 2
521   >1677      28S ribosomal RNA

>Feature gb|MW676292
<1    52         5.8S ribosomal RNA
53    522        internal transcribed spacer 2
523   >1679      28S ribosomal RNA

>Feature gb|MW676293
<1    52         5.8S ribosomal RNA
53    522        internal transcribed spacer 2
523   >1679      28S ribosomal RNA

>Feature gb|MW676294
<1    52         5.8S ribosomal RNA
53    522        internal transcribed spacer 2
523   >1679      28S ribosomal RNA

>Feature gb|MW676295
<1    52         5.8S ribosomal RNA
53    522        internal transcribed spacer 2
523   >1679      28S ribosomal RNA

>Feature gb|MW732930
<1    52         5.8S ribosomal RNA
53    520        internal transcribed spacer 2
521   >1677      28S ribosomal RNA

>Feature gb|MW732931
<1    52         5.8S ribosomal RNA
```

53	520	internal transcribed spacer 2
521	>1677	28S ribosomal RNA
