# Supplementary material for: Intragenomic sequence variations in the second internal transcribed spacer (ITS2) ribosomal DNA of the malaria vector Anopheles stephensi
Source: PLoS One. 2021 Jun 14;16(6):e0253173. doi: 10.1371/journal.pone.0253173 (PMC8202910; doi:10.1371/journal.pone.0253173)
Supplement: Attachment — Submitted filename: Response to Reviewers.docx [file pone.0253173.s002.docx]

**Response to Editor/Reviewers’ comments**

**Editor’s comment**

Discuss possible genomic reasons for the presence of intragenomic variation in rDNA. Can it be due to the presence of more than one copy of rDNA cluster in the genome of An. stephensi? For example, se the paper that shows the presence of the rDNA loci on both X and Y chromosomes, which may have different evolutionary trajectories. PMID: 25244985, PMCID: PMC4195908, DOI: 10.1186/s13059-014-0459-2

**Authors’ response:**

Thanks for the careful review of the manuscript and for suggesting to discuss the possible genomic reason for the presence of intragenomic variation in rDNA. We have incorporated a paragraph (**lines 227-240**; copied below), discussing the above. Please feel free to suggest if further edit is required for the improvement of the manuscript.

“The presence of intragenomic sequence variation in An. stephensi is indicative of less efficient concerted evolution acting on rDNA which can be due to the presence of multiple clusters of rDNA in the genome. Although the exact mechanisms of observed concerted evolution remain unclear, the homogenization of rDNA is believed to be due to a variety of genomic mechanisms of turnover such as unequal crossing over and gene conversion [41]. However, the concerted evolution is predicted to act less efficiently upon dispersed rDNA units [42] due to less frequent recombination on heterologous chromosomes [43]. Localization of rDNA clusters in An. stephensi through fluorescent in-situ hybridization (FISH) have revealed the presence of rDNA on both X and Y chromosomes [44], similar to Drosophila melanogaster [45] where interchromosomal exchanges are likely to be less efficient to homogenize rDNA. We noted intragenomic variations in ITS2 (non-coding rDNA) only but no evidence of intragenomic variation was found in coding rDNA sequenced in this study (partial 5.8S and d1-d3 domains of 28S-rDNA). Several reports cited by Keller et al. (2006) [46] have shown that often such intragenomic variation is restricted to the noncoding rDNA regions.”

**Reviewer #1:**

1-The manuscript is well written; however, it is unclear whether samples collected from different locations represent mysorensis, type or intermediate forms.

**Authors’ response:**

Mosquito samples used in this study were collected from the remote areas and preserved on site for molecular study. To classify their variant forms, we need to transport live mosquitoes, feed them on blood meal, allow them to lay their eggs individually and count ridges on egg floats, which we could not perform due to logistic reasons.

2-It is mentioned in the manuscript that "28S rDNA sequence can serve as molecular markers due to absence of obvious interagenomic variations", however, absence of interagenomic variations in 28S rDNA sequences are not shown in any figures.

**Authors’ response:**

Perhaps your question is related to the Figure displaying the alignment of DNA sequences. Alignment of the entire rDNA region we sequenced (1.7 kb) requires a lot of space (can be covered in 4 pages). We, therefore, preferred to show the alignment of ITS2 only to exhibit **polymorphic sites** and to **assign the base position numbering of each SNP/indel**. These numberings were used as identifier of each SNPs/indel in the manuscript. Because no SNP was observed in coding rDNA, I think there is no significance of displaying sequence alignment of these regions. The entire sequence of all haplotypes, however, is available at GenBank.

3-Line 159: Please correct the Accession number.

Also, correct the last two Accession numbers in Supplementary information S1 (Annotation Features of GenBank entries).

**Authors’ response:**

Thanks for the careful observation. We have corrected the accession numbers.

**Reviewer #2:**

Other molecular markers such as OBP1, COI, COII has been used for molecular identification of An. stephensi. The authors must explain why ITS2 and 28S rDNA were chosen, while OBP1 is one the most appropriate choice. I suggest to reflect this in the Introduction and Discussion sections.

**Authors’ response:**

We have mentioned the use of COI-mtDNA (COI and CytB) and OBP1 for the molecular identification of *An. stephensi* and also mentioned the proposal of the presence of three sibling species based on the polymorphism in the intronic region of this gene (**Gholizadeh et al., 2015; Firooziyan et al., 2018**; **lines 55-60**). Regarding the explanation why ITS2 and 28S rDNA was chosen (for the study), I would like to clarify that I have not chosen rDNA ‘for the identification’ but the purpose of this communication is to report intragenomic sequence variations in rDNA of *An. stephensi* and associated consequences on species delimitation and phylogenetic studies. This study by no means is proposing the use of rDNA as an alternative to other markers.
